# The Reality of Critical Cancer Patients in a Polyvalent Intensive Care Unit

**DOI:** 10.7759/cureus.13581

**Published:** 2021-02-26

**Authors:** Maria Teresa Neves, Inês Eiriz, Tiago C Tomás, Francisco Gama, Gabriela Almeida, Filipa B Monteiro, Tomás Lamas, Isabel Simões, Isabel Gaspar, Eduarda Carmo

**Affiliations:** 1 Oncology, Hospital São Francisco Xavier, Centro Hospitalar Lisboa Ocidental, Lisbon, PRT; 2 Medical Oncology, Hospital Prof. Doutor Fernando Fonseca, Lisbon, PRT; 3 Cardiology, Hospital Santa Cruz, Centro Hospitalar Lisboa Ocidental, Lisbon, PRT; 4 Polyvalent Intensive Care Unit, Hospital Egas Moniz, Centro Hospitalar Lisboa Ocidental, Lisbon, PRT

**Keywords:** oncological patients, hematological patients, intensive care unit stay

## Abstract

Background and objective

With the increasing incidence of cancer and the rise in the survival rates of cancer patients, more and more oncological candidates are being considered for admission to intensive care units (ICU). Several studies have demonstrated no difference in the outcomes of cancer patients compared to non-cancer patients. Our study aimed to describe and analyze the outcomes related to cancer patients in a polyvalent ICU.

Methods

We conducted a retrospective study of consecutive oncological patients admitted to a polyvalent ICU (2013-2017). Cox model and receiver operating characteristic (ROC) curve analysis were performed to analyze the results.

Results

A total of 236 patients were included in the study; the mean age of the patients was 53.5 ± 15.3 years, and 65% of them were male. The main cancer types were those related to the central nervous system (CNS; 31%), as well as gastrointestinal (18%), genitourinary (17%), and hematological (15%). Curative/diagnostic surgeries (49%) and sepsis/septic shock (17%) were the main reasons for admission. The Acute Physiology and Chronic Health Evaluation II (APACHE II) and Simplified Acute Physiology Score II (SAPS II) scores in hematological patients vs. solid tumors were as follows: 30 vs. 20 and 63 vs. 38, respectively (p<0.005). Vasopressors, invasive mechanical ventilation (IMV), and renal replacement therapy (RRT) were used more widely in hematological patients compared to solid-tumor patients. Length of stay was longer in hematological patients vs. solid-tumor patients (12.8 vs. 7 days, p=0.002). The median overall survival in hematological patients was one month and that in solid-tumor patients was 5.8 months (p<0.005). The survival rate at six months was better than described in the existing literature (48 vs. 32.4%).

Conclusion

Both SAPS II and APACHE II scores were reasonably accurate in predicting mortality, demonstrating their value in cancer patients.

## Introduction

The incidence of cancer is estimated to increase globally from 12.7 million new cases in 2008 to 22.2 million new cases by 2030 [1]. Similarly, in Portugal, we have seen a steady increase in cancer incidence at a constant rate of approximately 3% per year [2]. In parallel, there has been an increase in the survival rates of cancer patients due to earlier detection and the use of new therapeutic strategies [3].

The number of cancer patients has increased from one in 69 (1.4%) to one in 21 (4.8%) people in the last 30 years [4], and the five-year survival has increased by 67% for all types of cancers in the last two decades [4,5]. The combination of these factors has led to an increase in cancer patients being eligible for admission to intensive care units (ICU). Multicentric trials such as the Sepsis Occurrence in Acutely Ill Patients (SOAP) study [6] and the one conducted by Soares et al. [7] appear to demonstrate that there is no significant difference in the outcomes of cancer patients compared to non-cancer patients, which validates their admission to ICUs. Although some studies and clinical guidelines have been published on the criteria to be used for the admission of cancer patients to ICUs, there still remains an element of uncertainty about the precise indications for their admission and continuation or discontinuation of treatments [8-10].

With regard to decision-making pertaining to this subject, the short-term ICU prognosis and the long-term outcomes related to the oncological disease should always be considered [10]. Futile therapies that prolong suffering without achieving any clinical benefits should be avoided. Simultaneously, treatments preventing an avoidable death should not be suspended too early [11].

Thiéry et al. [12] conducted a study that evaluated all cancer patients for whom an ICU admission was requested during a one-year period. They assessed survival at 30 days and six months and found that in admitted patients, the survival rate at 30 days was 54.3% and 32.4% at six months. Interestingly, in patients who were refused ICU admission because they were considered too sick, 26% were still alive at 30 days and 16.7% at six months, while among patients who were refused admission because they were clinically well, the 30-day survival rate was 78.7% [12]. Apparently, there was a mismatch between the initial clinical evaluation and patient outcomes; in light of this, the need for a debate regarding the admission of cancer patients to ICUs is very important.

Against this backdrop, our study aimed to describe the characteristics and outcomes of cancer patients admitted to a polyvalent ICU in Portugal.

Results of this study were presented as a poster in ESMO Congress 2019 in Barcelona in September 2019 and were subsequently published as an abstract in the journal Annals of Oncology, volume 30, supplement 5, October 2019 (DOI: 10.1093/annonc/mdz265.009).

## Materials and methods

This was a retrospective single-center analysis conducted in a polyvalent ICU at the Egas Moniz Hospital in Lisbon, Portugal. This study was performed according to the Declaration of Helsinki. Clinical data relating to 254 oncological patients admitted in the hospital's ICU between January 2013 and December 2017 were obtained; 16 patients were excluded because the oncological disease was not considered to be active in them (disease in complete remission for at least two years). Two patients were excluded because they had cancer of an unknown primary site. Hence, 236 patients were selected for the analysis.

Epidemiological and clinical data collected from patients’ medical records were as follows: sex, age, time of ICU admission, type of cancer, cause of ICU admission, the Acute Physiology and Chronic Health Evaluation II (APACHE II) and Simplified Acute Physiology Score II (SAPS II) scores, therapeutic interventions during ICU stay [use of vasopressors, mechanical ventilation, or renal replacement therapy (RRT) for more than 24 hours], length of ICU stay, ICU and in-hospital mortality, survival at one month, six months, five years, and overall survival (OS). OS was defined as the period of time from admission in ICU until final analysis or death, whichever was earlier. Organ support was only considered if used for more than 24 hours, thereby excluding patients who were submitted to mechanical ventilation for airway protection in a surgical context and those who were previously on chronic dialysis.

Sub-analysis excluding patients with solid central nervous system (CNS) tumors was performed since all of them were admitted after an elective procedure (e.g., tumor resection) and not due to disease-related decompensation or progression.

Continuous variables were tested for normality of distribution by using the Shapiro-Wilk test and were reported and analyzed appropriately thereafter. Categorical variables were compared by chi-square statistics or the Fisher's exact test. Mann-Whitney U test was used in cases of abnormal distribution. Receiver operating characteristic (ROC) curves analyzing APACHE II and SAPS II score sensitivity were generated using overall death as the endpoint. Multivariable analysis with the Cox regression test was also performed to identify significant predictors of the outcome. All the analyses were considered significant at a two-tailed p-value of <0.05. The SPSS Statistics version 22.0 (IBM, Armonk, NY) was used to perform all statistical evaluations.

## Results

A total of 236 patients were included in our retrospective study, and they were selected from among 1,400 ICU admissions during the study period. The baseline characteristics of enrolled patients are listed in Table 1. The mean age of the patients was 53.5 ± 15.3 years, and 65.7% of them were males. The main types of cancer were those related to CNS (n=72; 30.5%), as well as gastrointestinal (n=42; 17.8%), genitourinary (n=40; 16.9%), and hematological (n=35; 14.8%). Major reasons for ICU admission were surgeries with curative or diagnostic intent (n=115; 48.7%) and severe sepsis or septic shock (n=41; 17.4%). Regarding the need for organ support, most patients required invasive mechanical ventilation (IMV) (n=77; 32.6%), non-invasive ventilation (n=20; 8.5%), vasopressors (n=71; 30.1%), and RRT (n=33; 14%).

ICU evaluation scores used regularly at admission were APACHE II (median: 17; minimum: 2; maximum: 50) and SAPS II (median: 34; minimum: 0; maximum: 104). The median length of stay in the ICU was three days [interquartile range (IQR): 5; minimum: 0; maximum: 46].

Outcomes and univariable analysis

ICU mortality was 16.1% (n=38) and in-hospital mortality was 14.4% (n=34). ICU mortality in patients who required RRT was 66.7% (n=22); ICU mortality in those who required non-invasive ventilation, IMV, and vasopressors was as follows: 60% (n=12), 41.6% (n=32), and 43.7% (n=31), respectively. In-hospital mortality in patients who required vasopressors, IMV, non-invasive ventilation, and RRT was as follows: 22.5% (n=15), 19.5% (n=15), 15% (n=3), and 12.1% (n=4), respectively.

**Figure 1:**
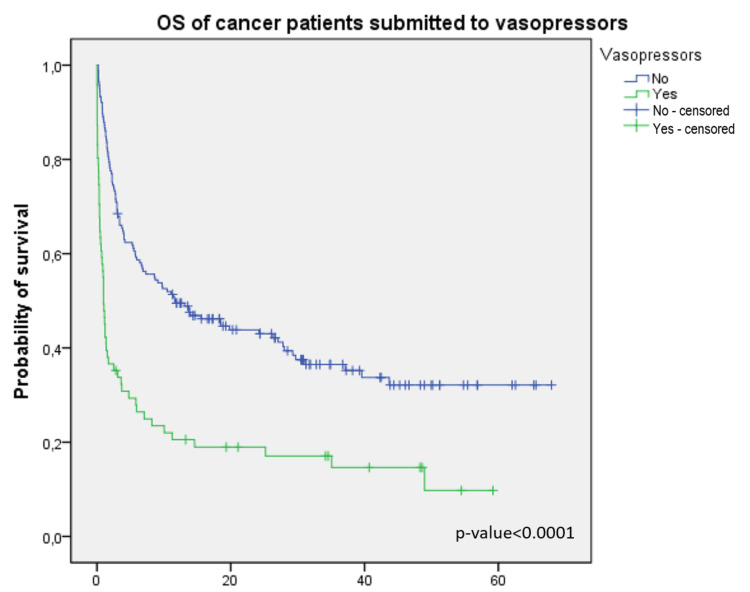
Overall survival in cancer patients submitted to vasopressors OS: overall survival

**Figure 2:**
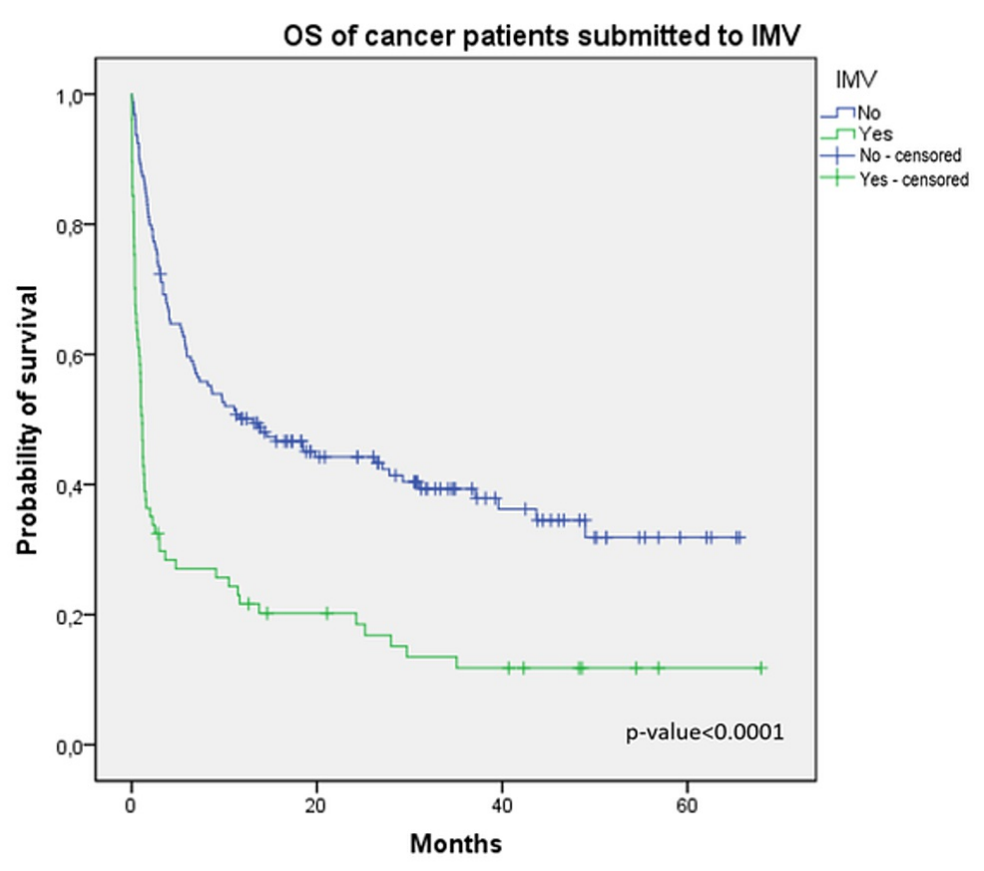
Overall survival in cancer patients submitted to invasive mechanical ventilation OS: overall survival; IMV: invasive mechanical ventilation

Median OS was 5.7 months, with a survival rate at six months of 48%. Median OS in patients who required vasopressors was 0.9 vs. 11.6 months (Figure 1); the median OS in patients who required IMV (Figure 2), non-invasive ventilation, and RRT was as follows: 1.1 vs. 13.1 months, 1 vs. 7.3 months, and 0.8 vs. 9.7 months, respectively (p<0.001).

Univariable comparisons of clinical characteristics and outcomes of solid tumors (except CNS) and hematological cancer patients are presented in Table 2. Vasopressors, IMV, non-invasive ventilation, and RRT were used more widely in hematological patients rather than solid-tumor patients (71.4% vs. 34.9%, p<0.001; 62.8% vs. 38.8%, p=0.013; 28.6% vs. 7.8%, p=0.002; 45.7% vs. 13.2%, p<0.001, respectively). Sepsis and septic shock were more prevalent in hematological patients when compared with solid-tumor patients (45.7% vs. 19.4%, p<0.001), and so were respiratory failures (31.4% vs. 11.6%, p<0.001). In solid-tumor patients, surgeries were the main reasons for ICU admission (curative or diagnostic in 38.7% and palliative in 21.7%).

**Figure 3:**
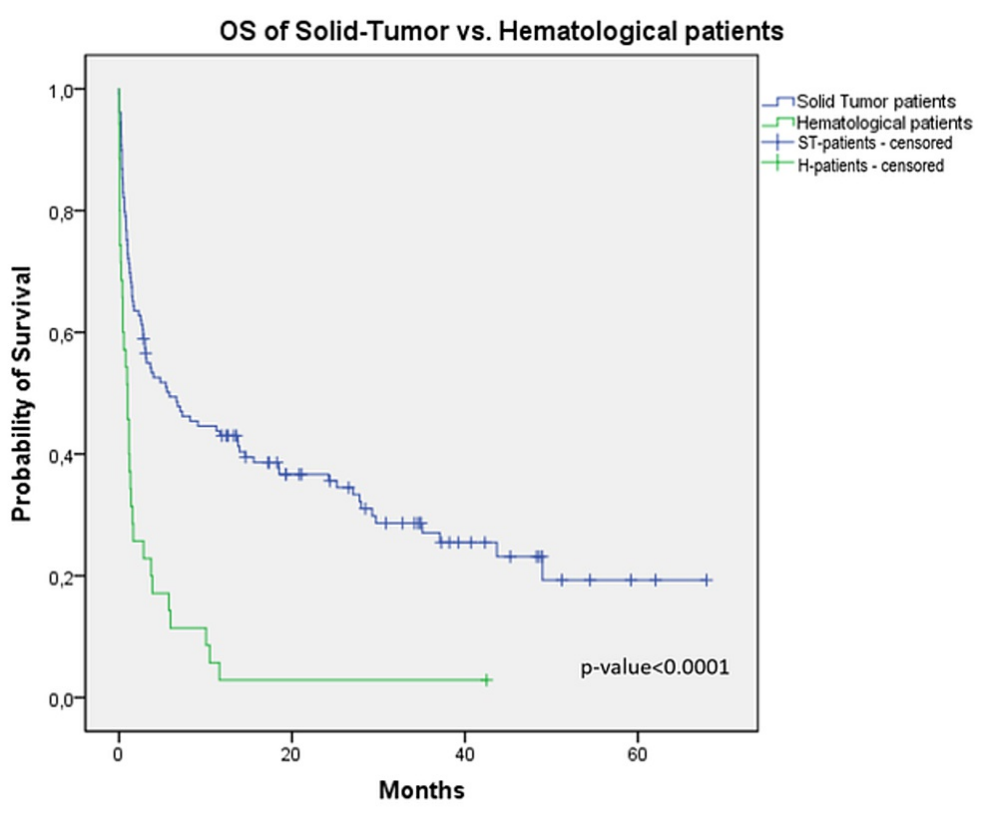
Overall survival in solid-tumor patients versus hematological patients OS: overall survival

**Figure 4:**
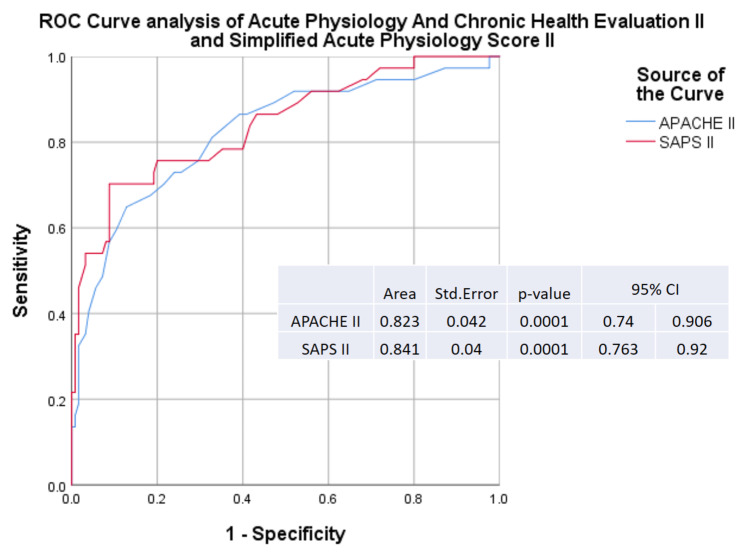
ROC curve analysis of SAPS II and APACHE II ROC: receiver operating characteristic; SAPS II: Simplified Acute Physiology Score II; APACHE II: Acute Physiology and Chronic Health Evaluation II

Median APACHE II and SAPS II scores were higher in hematological patients (30.1 vs. 19.9, p<0.001; 62.7 vs. 38.4, p<0.001, respectively) when compared with solid-tumor patients. Duration of ICU stay was also more prolonged in hematological patients (12.8 vs. 7 days, p=0.002); ICU mortality was higher in hematological patients (57.1% vs. 14%, p<0.001). A better OS was documented in patients with solid malignancies (5.8 vs. one month; 95% CI: 2.5-9.1, p<0.001) (Figure 3). APACHE II had 71.8% sensitivity and 60% specificity in predicting mortality (95% CI: 0.74-0.91, p<0.0001), and SAPS II had a sensitivity of 77.6% and specificity of 71% (95% CI: 0.76-0.92, p<0.0001), according to the ROC curve (Figure 4). The area under the curve (AUC) for APACHE II was 0.82 ± 0.042, and that for SAPS II was 0.84 ± 0.04 (Figure 4).

Multivariable analysis

There was a higher risk of death in patients with hematological cancer in comparison with solid-tumor patients (adjusted odds ratio: 4.08, 95% CI: 3.6-18.9, p<0.001). In our population, use of organ support was associated with a higher risk of death: vasopressors (adjusted odds ratio: 10.4, 95% CI: 7.2-42.6, p<0.001); IMV (adjusted odds ratio: 10.9, 95% CI: 7.1-46.1, p<0.001); non-invasive ventilation (adjusted odds ratio: 5, 95% CI: 4.1-29.3, p<0.001), and RRT (adjusted odds ratio: 8.4, 95% CI: 9.6-56.7, p<0.001).

**Table 1:** Baseline demographics and characteristics of cancer patients in ICU ICU: intensive care unit; CNS: central nervous system; APACHE II: Acute Physiology and Chronic Health Evaluation II; SAPS II: Simplified Acute Physiology Score II

Variables	Values (n=236)	
Age, years (mean)	53.5	
Males, n/%	155	65.7
Types of cancer	N	%
Genitourinary	40	16.9
Bladder	11	4.7
Kidney	13	5.5
Urothelial	4	1.7
Prostate	6	2.5
Testicle	1	0.4
Cervix	1	0.4
Ovarian	2	0.8
Vulvar	2	0.8
Gastrointestinal	42	17.8
Colon	17	7.2
Rectal	6	2.5
Esophagus	9	3.8
Stomach	10	4.2
Breast	6	2.5
Head and neck	19	8.1
Lung	21	8.9
Bone	1	0.4
CNS	72	30.5
Hematological	35	14.8
Major reason for ICU admission	N	%
Respiratory failure	26	11
Sepsis/septic shock	41	17.4
Acute renal failure	3	1.3
Chemotherapy toxicity	4	1.7
Curative/diagnostic surgery	115	48.7
Palliative surgery	37	15.7
Disease progression	10	4.2
ICU therapeutic interventions	N	%
Invasive mechanical ventilation	77	32.6
Non-invasive ventilation	20	8.5
Vasopressors	71	30.1
Renal replacement therapy	33	14
ICU evaluation scores (median)		
APACHE II	17	
SAPS II	36	

**Table 2:** Univariable analysis for solid versus hematological cancer patients (excluding central nervous system tumors) ICU: intensive care unit; APACHE II: Acute Physiology and Chronic Health Evaluation II; SAPS II: Simplified Acute Physiology Score II

Variables	Solid tumors (n=129)		Hematological (n=35)		P-value
Age, years (median)	67.5		65.8		0.52
Males, n/%	84	65.1	24	68.6	0.84
Major reason for ICU admission	N	%	N	%	
Respiratory failure	15	11.6	11	31.4	<0.001
Sepsis/septic shock	25	19.4	16	45.7	<0.001
Acute renal failure	3	2.3	0	0	<0.001
Chemotherapy toxicity	0	0	4	11.4	<0.001
Curative/diagnostic surgery	50	38.7	2	5.7	<0.001
Palliative surgery	28	21.7	2	5.7	<0.001
Disease progression	8	6.2	0	0	<0.001
ICU therapeutic interventions	N	%	N	%	
Invasive mechanical ventilation	50	38.8	22	62.8	0.013
Non-invasive ventilation	10	7.8	10	28.6	0.002
Vasopressors	45	34.9	25	71.4	<0.001
Renal replacement therapy	17	13.2	16	45.7	<0.001
ICU evaluation scores (median)					
APACHE II	19.9		30.1		<0.001
SAPS II	38.4		62.7		<0.001
Outcomes					
Length of ICU stay, days (median)	7		12.8		0.002
	N	%	N	%	
ICU mortality	18	14	20	57.1	<0.001
In-hospital mortality	23	17.8	6	17.1	1
Out-of-hospital mortality	8	6.2	5	14.3	0.11

## Discussion

Almost 17% of patients admitted in our ICU during the five-year study period were cancer patients, and nearly one-third of these patients had CNS cancers (n=72; 30.5%), followed by gastrointestinal (n=42; 17.8%), genitourinary (n=40; 16.9%), and hematological (n=35; 14.8%). This could be attributed to this hospital being a neurosurgery reference center. This may also explain the fact that the main reason for ICU admissions was surgeries with curative or diagnostic intent (n=115; 48.7%), which is in line with the findings of other studies [1]. Severe sepsis or septic shock (n=41; 17.4%) and palliative surgery (n=37; 15.7%) were other important causes for admission. The use of mechanical ventilation was required in almost one-third of patients and vasopressors in 30.1%. These results are in line with 28.4% of admissions being caused by respiratory failure and septic shock.

ICU mortality was 16.1%, and in-hospital mortality was 14.4%, which is consistent with the findings of previous reports from other European ICUs [13]. Decreased mortality rate could be due to two factors: firstly, the development of more potent and targeted anti-tumor therapies, advances in the standard strategies for determining indications and supportive care, as well as progress in the prevention of organ dysfunction; secondly, with a deeper understanding of the pathophysiological mechanisms in organ dysfunction, intensive care has improved survival rate among patients with critical illness by constantly renewing strategies for survival of patients with sepsis, hemodynamic monitoring, mechanical ventilation, nutrition support, sedation, and analgesia [1]. However, as patients with solid tumors after elective surgery were the main group admitted to our ICU, low mortality in our study can be related to that condition. ICU mortality in patients who required RRT was 66.7%; ICU mortality in patients who required non-invasive ventilation, IMV, and vasopressors was as follows: 60%, 41.6%, and 43.7%, respectively. The higher mortality in patients submitted to non-invasive ventilation when compared with IMV can be attributed to non-invasive ventilation being the therapeutic ceiling in fragile patients or in those who have a poor prognosis and hence associated with higher mortality. The use of IMV, vasopressors, and RRT were important predictors of mortality in our study and they appear to be relevant variables influencing the median OS. These measures of organ support were used in critically ill patients, which indicates that high mortality was due to severe disease and not organ support measures themselves. 

In our study, the median OS was 5.7 months, with a survival rate at six months of 48%, which is better than what is described in the existing literature [12,14]. Six months can offer a patient the opportunity to receive anti-cancer treatment after ICU treatment. Active treatment in the ICU could be more important than many anti-cancer therapies if it offers the possibility of prolonging survival with good quality of life for more than three months [1]. The endpoint of therapy in patients with advanced-stage cancer differs from that in patients without active neoplastic disease. The concern should not be survival rate only but also the quality of life and long-term survival. Triage decisions solely based on the type of cancer are hence not justified [1]. Intensivists often need to make quick decisions based on little or inconclusive information. Sometimes, we may find a high hospital survival rate among a small number of patients for whom an agreement to limit the care was not achieved [15]. Rapid selection depending on unreliable triage criteria will inevitably lead to undertreatment and unnecessary death in selected patients [16].

The need to maintain a balance between reasonable hope of benefit and excessive burden on the family or community urgently requires an effective oncology critical scoring system and risk factors analysis to broaden ICU admission criteria for patients with cancer [3]. APACHE II and SAPS II are the most commonly used scoring systems in ICUs in Portugal, which are based on multiple logistic regression equations describing abnormalities in physiological variables during the first 24 hours of ICU admission. These calculation methods result in predicted mortality. In our population, the ROC curve for SAPS II and APACHE II had good sensitivity and specificity in predicting mortality. These scores enable the initial assessment of the patient in ICU admission and could be used as a tool in monitoring clinical evolution and management of expectations throughout hospitalization. Its applicability is very important in cancer patients.

When comparing hematological patients with solid-tumor patients, sepsis and septic shock were more prevalent in the former (45.7% vs. 19.4%, p<0.001), and so was respiratory failures (31.4% vs. 11.6%, p<0.001), due to cancer-related issues and more aggressive treatment complications, such as severe and more prolonged medullary aplasia [17]. In solid-tumor patients, surgeries were the main reason for ICU admission (curative or diagnostic in 38.7% and palliative in 21.7%). Hematological patients needed more IMV than patients with solid tumors (62.8% vs. 38.8%), and the same was the case with vasopressors (71.4% vs. 34.9%) and RRT (45.7% vs. 13.2%). Hematological patients had higher median APACHE II and SAPS II scores as well as ICU mortality rates, probably revealing a more ill population at admission. These results could be related to the main causes of admission of these patients (respiratory failure and sepsis/septic shock).

Our study has several limitations: it was a retrospective study conducted at a single cancer center, and the small size of the sample prevented us from investigating the characteristics of critical illness in patients with different types of solid cancer and the effect of ambulatory chemotherapy. Besides, in our population, there was a high prevalence of elective hospitalizations after surgery. However, this is the first report about clinical characteristics, prognosis, and risk factors of critically ill patients with solid and hematological tumors in a Portuguese ICU.

## Conclusions

In our study, the survival rate at six months was better than what is described in the literature. Prolonged ICU stay was associated with a worse prognosis, and so was the use of supportive therapies. A better OS was documented in solid-tumor patients when compared to hematologic patients. Both SAPS II and APACHE II scores were reasonably accurate in predicting mortality, demonstrating their value in cancer patients. It is essential for people who work in ICUs to be aware of the fact that the goal of treatment may shift from curative or supportive therapy to end-of-life care.
